# Phase-aberration-corrected coherence factor beamforming for photoacoustic computed tomography

**DOI:** 10.1117/1.JBO.31.10.106003

**Published:** 2026-10-09

**Authors:** Xiali Gao, Zhichao Zheng, Zhibo Xiao, Xu Sun, Nuomin Zhang, Yang Xiao, Hao Huang, Xuran Zhou, Fan Meng, Xuanhao Wang, Ruimin Chen, Junhui Shi

**Affiliations:** a Zhejiang University, College of Biomedical Engineering and Instrument Science, Hangzhou, China; b Zhejiang Lab, Hangzhou, China; c Jilin University, School of Mathematics, Department of Computational Mathematics, Changchun, China; d Harbin Institute of Technology, School of Aeronautics, Control Theory and Engineering, Harbin, China; e State Key Laboratory of Ocean Sensing, Hangzhou, China

**Keywords:** photoacoustic computed tomography, coherence factor, volumetric phase aberration, 3D beamforming, vascular imaging

## Abstract

**Significance:**

Coherence factor (CF) beamforming can suppress sidelobes in photoacoustic computed tomography, but phase aberration can make genuine absorbers appear incoherent and cause conventional CF to downweight real signals.

**Aim:**

We aim to reduce photoacoustic volume tomography phase-aberration-induced loss of true signals in conventional coherence-factor reconstruction and validate the method using phantom and *in vivo* experiments.

**Approach:**

PAC-CF extends an ellipsoidal two-region time-of-flight correction to the angle-weighted channel contributions used for both coherent backprojection and final conventional energy-ratio CF evaluation. The ROI-optimized effective internal sound speed is selected from multiframe PA receive data by maximizing the mean CF within a fixed region of interest.

**Results:**

Simulations showed that conventional CF at the true source dropped to 0.00794 at a 170  m/s sound-speed difference and to ∼0.00039 to 0.00042 at larger sound-speed differences, whereas PAC-CF preserved coherence and localization. The four-method phantom ablation showed that combining two-region TOF correction with final CF weighting produced the most concentrated wire responses and strongest background suppression, whereas mouse experiments showed recovery of vascular features suppressed by conventional CF.

**Conclusions:**

By extending two-region time-of-flight correction to the channel contributions evaluated by the final CF weight, PAC-CF reduces the erroneous suppression of true signals caused by propagation-model mismatch and provides a practical PA-only approach for coherence-weighted three-dimensional PACT.

## 1 Introduction

Photoacoustic (PA) imaging detects ultrasound waves generated by pulsed optical absorption, combining optical absorption contrast with acoustic-resolution imaging at depths beyond purely optical microscopy.^1^^–^^4^ Photoacoustic computed tomography (PACT) records these waves over a broad aperture for volumetric reconstruction.^5^^,^^6^ Delay-and-sum (DAS) backprojection remains common in hemispherical-array PACT because it is simple and computationally efficient,^7^^–^^9^ but it retains sidelobes, streak artifacts, and off-axis background.^10^^–^^12^ Coherence factor (CF) weighting suppresses these artifacts by comparing coherent and incoherent aperture energy; when the delay law is correct, true absorber signals add coherently and are preserved.^13^^,^^14^

The weakness of CF is that it judges coherence only after delay compensation has already been applied. If the assumed time-of-flight (TOF) model is wrong, the delayed samples from a real absorber are no longer phase aligned.^15^^,^^16^ Tissue, skin, fat, organ parenchyma, and coupling water have different sound speeds, so PA waves accumulate detector-dependent phase aberrations before reaching the array.^17^^–^^20^ Conventional CF cannot distinguish this phase-aberrated true signal from incoherent background and may therefore suppress structures that should be retained.

Previous studies have used PA coherence both to select a homogeneous effective sound speed and to weight the final two-dimensional beamformed image.^21^ Other full-ring dual-modal studies used pulse-echo US coherence to select either a mean sound speed or dual-region sound speeds for subsequent PA reconstruction.^22^^,^^23^ Separately, ellipsoidal dual-speed models have been incorporated into three-dimensional hemispherical UBP reconstruction to account for the sound-speed difference between tissue and the coupling medium.^24^^,^^25^ Together with prior autofocus, speed-compensated, full-wave, and learning-based approaches,^26^^–^^32^ these studies establish the value of coherence-guided sound-speed selection, CF weighting, and dual-region propagation correction. The present work addresses the methodological gap between these lines of research by applying ellipsoidal two-region correction to the same angle-weighted channel contributions used for final voxel-wise conventional CF evaluation in three-dimensional hemispherical PA-only PACT.

Here, we present phase-aberration-corrected coherence factor (PAC-CF) beamforming for PACT. The contribution does not lie in the ellipsoidal two-region model, coherence-guided sound-speed search, or conventional CF individually; it lies in extending the corrected TOF to the angle-weighted channel contributions used for final voxel-wise conventional CF evaluation in three-dimensional hemispherical PA-only PACT. Coherence has two connected but distinct roles: the ROI- and frame-averaged CF selects an effective internal sound speed entirely from PA receive data, whereas the voxel-wise CF weights the final image after partial phase alignment. This formulation requires neither pulse-echo US data nor an independently measured sound-speed map. Simulations, crossed-wire phantom data, and *in vivo* mouse vascular data show that PAC-CF preserves the artifact-suppression benefit of CF while recovering true structures that conventional CF can attenuate (Table 1).

**Table 1:** Comparison of the reconstruction variants evaluated in this study and closely related sound-speed modeling and coherence-weighting strategies.

Method/study	Dimensionality/data	Sound-speed model	Role of CF	Final PA reconstruction
Yoon et al.^21^	2D linear-array PA	Homogeneous effective speed	PA CF for speed selection and final weighting	CF-weighted DAS
VF-USPACT^22^	2D full-ring US/PA	Homogeneous mean speed	US CF for speed selection	Weighted DAS
ADS-USPACT^23^	2D full-ring US/PA	Two-region dual speed	US CF for speed selection	Dual-speed weighted DAS
3D-PACT^24^	3D hemispherical PA	Half-ellipsoid dual speed	Not used	Dual-speed UBP
3D-PanoPACT^25^	3D hemispherical PA	Ellipsoid dual speed	Not used	Dual-speed UBP
DAS (this work)	3D hemispherical PA-only	Single-speed TOF model	Not used	DAS
Conventional CF (this work)	3D hemispherical PA-only	Single-speed TOF model	Final PA weighting only	DAS with final PA energy-ratio CF weighting
PAC-DAS (this work)	3D hemispherical PA-only	Ellipsoidal two-region effective-speed model	PA CF for effective-speed selection only	Two-region TOF-corrected DAS
PAC-CF (this work)	3D hemispherical PA-only	Ellipsoidal two-region effective-speed model	PA CF for effective-speed selection and final weighting	Two-region TOF-corrected DAS with final PA energy-ratio CF weighting

## 2 Materials and Methods

### 2.1 Overview of the PAC-CF Workflow

The PAC-CF workflow is summarized in Fig. 1. Figure 1(a) illustrates the PACT acquisition process, in which pulsed laser illumination generates acoustic waves that are detected by a hemispherical transducer array. Figure 1(b) contrasts conventional CF with PAC-CF: conventional CF evaluates coherence after homogeneous-delay compensation, whereas PAC-CF first applies the ellipsoidal two-region TOF correction and then evaluates and applies the same energy-ratio CF. Figures 1(c) and 1(d) show the two-region target model and representative sinogram. The coupling-water sound speed is measured from temperature, an initial reconstruction provides spatial guidance [Fig. 1(e)], and an ellipsoidal prior is placed around the target region [Fig. 1(f)]. The effective internal sound speed is selected by maximizing mean CF within the fixed optimization ROI; the CF-score curve shown in Fig. 1(g) is the actual search curve obtained from the mouse experiment. Final reconstruction is then performed with the two-region TOF model [Fig. 1(h)].

**Fig. 1:**
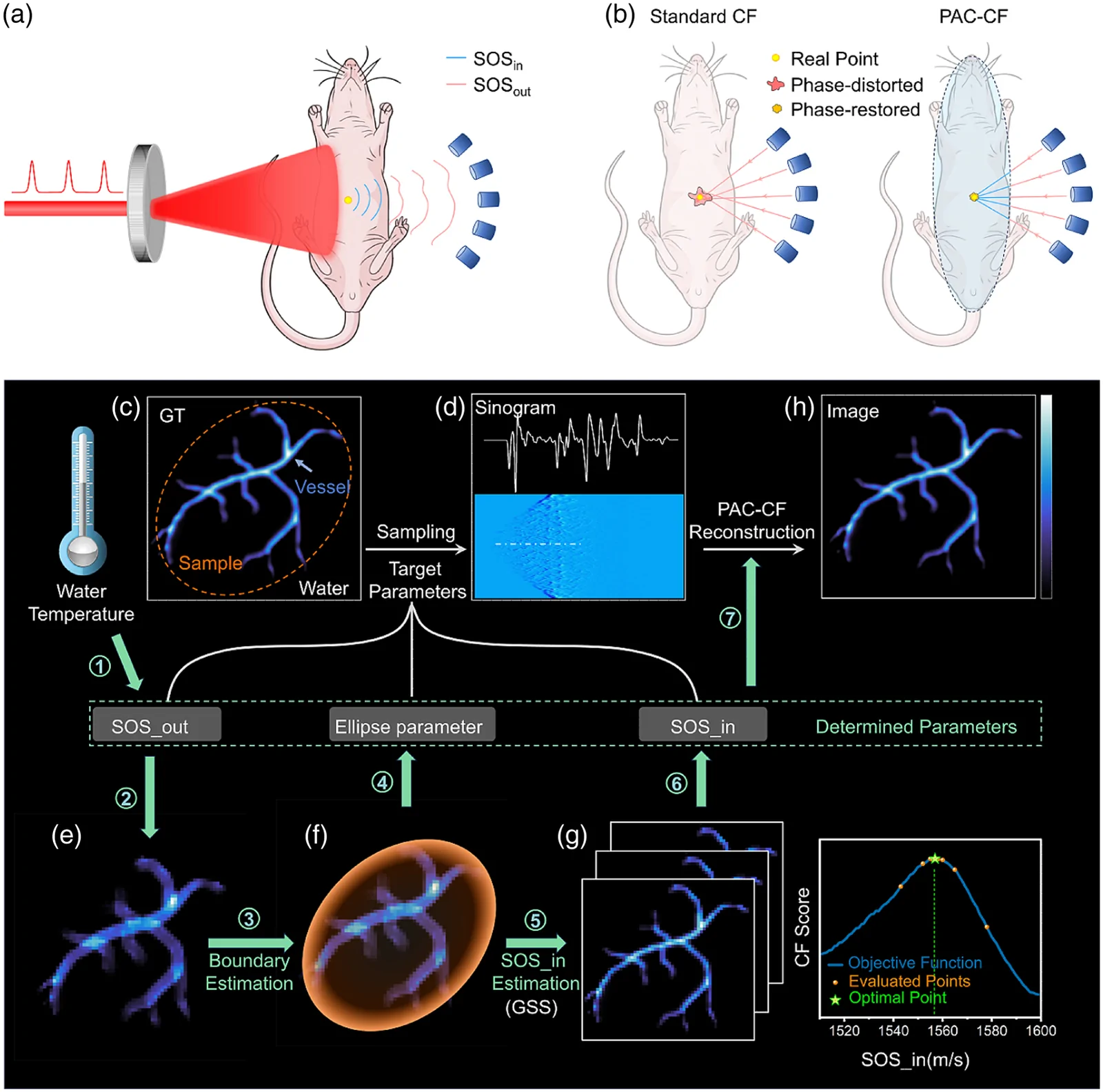
Schematic of the PAC-CF framework. (a) PACT data acquisition. (b) Comparison between conventional homogeneous-delay CF and PAC-CF, which evaluates coherence after two-region TOF correction. (c) Ground-truth sample model containing a target region and surrounding coupling medium. (d) Representative photoacoustic sinogram. (e) Initial low-resolution reconstruction using the background speed determined from water temperature. (f) Ellipsoidal prior enclosing the target. (g) Coherence-guided selection of the effective internal sound speed, where the objective is the mean CF within the selected ROI; the plotted CF-score curve is the actual search curve from the mouse experiment. (h) Final PAC-CF reconstruction using the optimized two-region TOF model.

### 2.2 Conventional DAS and Coherence-Factor Weighting

Let $s_{n}(t)$ denote the radio-frequency photoacoustic signal at time t recorded by detector element n, let r denote an image voxel, and let $r_{n}$ denote the position of the n’th detector. In homogeneous DAS reconstruction, the channel delay $\tau_{n}(r)$ is computed from the Euclidean distance between r and $r_{n}$ divided by a single assumed sound speed $v_{0}$. The DAS image is calculated as

$$I_{\mathrm{DAS}}(r)=\frac{\overset{N}{\underset{n=1}{\sum }}w_{n}(r)s_{n}(\tau_{n}\left(r\right))}{\overset{N}{\underset{n=1}{\sum }}w_{n}(r)}.$$ (1)

For CF weighting, the angle-weighted channel contribution is denoted $y_{n}(r)=w_{n}(r)s_{n}(\tau_{n}(r))$, where $w_{n}(r)$ is the angular-geometric weight used consistently in DAS, CF, PAC-DAS, and PAC-CF. The coherence factor follows the conventional energy-ratio definition, in which the squared coherent aperture sum is normalized by the incoherent channel energy

$$C(r)=\frac{|\overset{N}{\underset{n=1}{\sum }}y_{n}(r){|}^{2}}{N\overset{N}{\underset{n=1}{\sum }}|y_{n}(r){|}^{2}+\varepsilon },$$ (2)

where N is the number of detector elements and ε is a small numerical constant preventing division by zero. The CF-weighted reconstruction is then written as

$$I_{\mathrm{CF}}(r)=I_{\mathrm{DAS}}(r)C(r).$$ (3)

Conventional CF and PAC-CF use the same energy-ratio CF definition and apply the resulting factor once; their difference lies only in the delay model used to obtain the channel samples. Conventional CF uses the homogeneous delay $\tau_{n}(r)$ as DAS. If this delay does not compensate the true propagation time, the samples $y_{n}(r)$ are not phase-aligned even for a real absorber, and C(r) can be artificially low.

#### 2.2.1 PAC-CF with two-region TOF correction

PAC-CF modifies the delay law used before CF is evaluated. Instead of treating all propagation paths as if they travel at a single homogeneous speed, it uses a compact ellipsoidal two-region prior to estimate the detector-dependent delay error. In this study, the target is approximated by an ellipsoidal region inside the surrounding coupling medium, which provides a simple way to estimate the path portions inside and outside the target for each voxel-detector pair. This prior is not introduced as a new acoustic propagation theory, and it is used only to reduce phase aberration before the CF energy ratio is computed.

In the reconstruction coordinate system, the implemented prior is an axis-aligned ellipsoid; no rotational degrees of freedom are used. Let its center be $r_{c}=(x_{c},y_{c},z_{c})$ and its semi-axis lengths be (a,b,c). A voxel position $r=(x_{c},y_{c},z_{c})$ is referenced to this ellipsoid, whose boundary is

$$\frac{{\left(x-x_{c}\right)}^{2}}{a^{2}}+\frac{{\left(y-y_{c}\right)}^{2}}{b^{2}}+\frac{{\left(z-z_{c}\right)}^{2}}{c^{2}}=1.$$ (4)

Points for which the left-hand side of Eq. (4) is less than or equal to 1 are treated as inside the target region. For detector n at $r_{n}=(x_{n},y_{n},z_{n})$, the voxel-detector path is modeled as the straight segment

$$p(t)=r_{n}+t(r-r_{n}),\;0\leq t\leq 1,$$ (5)

where $d=r-r_{n}=(d_{x},d_{y},d_{z})$. Substitution of Eq. (5) into Eq. (4) gives a quadratic equation in t

$$At^{2}+Bt+C=0\;A=\frac{{d_{x}}^{2}}{a^{2}}+\frac{{d_{y}}^{2}}{b^{2}}+\frac{{d_{z}}^{2}}{c^{2}}\;B=2[\frac{(x_{n}-x_{c})d_{x}}{a^{2}}+\frac{(y_{n}-y_{c})d_{y}}{b^{2}}+\frac{(z_{n}-z_{c})d_{z}}{c^{2}}]\;C=\frac{{\left(x_{n}-x_{c}\right)}^{2}}{a^{2}}+\frac{{\left(y_{n}-y_{c}\right)}^{2}}{b^{2}}+\frac{{\left(z_{n}-z_{c}\right)}^{2}}{c^{2}}-1.$$ (6)

The discriminant and candidate intersection parameters are

$$\Delta =B^{2}-4AC,\;t_{1,2}=\frac{-B\pm \sqrt{\Delta }}{2A}.$$ (7)

Only the portion of the infinite line that lies on the physical segment 0≤t≤1 is retained. For Δ≥0, define the clipped entry and exit parameters and corresponding path lengths as

$$t_{-}=\max(0,\min(t_{1},t_{2})),\;t_{+}=\min(1,\max(t_{1},t_{2}))\;L(r,n)=\sqrt{{d_{x}}^{2}+{d_{y}}^{2}+{d_{z}}^{2}}\;L_{\text{in}}(r,n)=L\cdot \max(0,t_{+}-t_{-}),\;L_{\text{out}}(r,n)=L-L_{\text{in}}(r,n).$$ (8)

Equation (8) also covers voxels outside the ellipsoid. If the segment does not cross the ellipsoid (Δ<0, tangency, or no positive clipped interval), $L_{\text{in}}=0$ and $L_{\text{out}}=L$. For an exterior voxel reached after two crossings, only the interval between the clipped roots is assigned to the internal region, whereas the detector-to-entry and exit-to-voxel portions are both included in $L_{\text{out}}$. For a voxel inside the ellipsoid, the second root is clipped to $t_{+}=1$, so $L_{\text{in}}$ extends from the entry point to the voxel. All detector elements are outside the ellipsoid in the present geometry. The model assumes straight-line propagation and neglects refraction, diffraction, and curved-ray effects; it is therefore a compact two-region TOF correction rather than a reconstruction of the complete sound-speed field.

With $v_{\mathrm{in}}$ and $v_{\mathrm{out}}$ denoting the effective internal and external sound speeds, respectively, the corrected TOF is

$$T_{\mathrm{pac}}(r,n)\;=\;\frac{L_{\text{out}}(r,n)}{v_{\text{out}}}\;+\;\frac{L_{\text{in}}(r,n)}{v_{\text{in}}}.$$ (9)

The corrected delay $T_{\mathrm{pac}}(r,n)$ is then used to sample the channel data, producing $y_{n,\mathrm{pac}}(r)=w_{n}(r)s_{n}(T_{\mathrm{pac}}(r,n))$. The PAC-CF coherence factor $C_{\mathrm{pac}}(r)$ is computed using the same energy-ratio definition as Eq. (2), with $y_{n,\mathrm{pac}}(r)$ replacing $y_{n}(r)$. Thus, PAC-CF is not a different CF formula; it changes the phase state of the data on which the CF decision is made.

$$I_{\mathrm{PAC}-\mathrm{CF}}(r)=I_{\mathrm{DAS}\_\mathrm{pac}}(r)C_{\mathrm{pac}}(r),$$ (10)

where $I_{\mathrm{DAS}\_\mathrm{pac}}(r)$ is the coherent backprojection term formed with $T_{\mathrm{pac}}(r,n)$ and $C_{\mathrm{pac}}(r)$ is the phase-corrected CF. This formulation makes the coherence judgment after phase-aberration correction rather than under the homogeneous-delay assumption.

In PAC-CF, the two-region TOF correction is not limited to the coherent backprojection term; the resulting angle-weighted channel contributions are also used to calculate the final voxel-wise energy-ratio CF. The ROI- and frame-averaged CF used for sound-speed selection and the voxel-wise CF used for final image weighting share the same conventional energy-ratio definition but serve different purposes.

#### 2.2.2 Coherence-guided effective internal sound-speed selection

The ellipsoidal two-region prior contains an external speed $v_{\text{out}}$ and an effective internal sound speed $v_{\text{in}}$. The external speed was determined from the coupling-water temperature measured using a UNI-T UT320D thermometer with a K-type thermocouple probe. The meter has a display resolution of 0.1°C and a manufacturer-specified accuracy of ±(0.5% of the reading +1°C) over the relevant temperature range, excluding thermocouple-probe error. The water sound speed was calculated using the k-wave waterSoundSpeed implementation of the fifth-order Marczak polynomial;^33^ the corresponding curve and measured-temperature points are shown in Fig. S3 in the Supplementary Material. The effective internal sound speed was treated as a scalar parameter defining the corrected TOF within the target region. For a selected region of interest (ROI), denoted Ω, the objective function was the average PAC-CF value over the fixed ROI and all K frames

$$J(v_{\text{in}})=\frac{1}{K}\overset{K}{\underset{k=1}{\sum }}[\frac{1}{\left|\Omega \right|}\underset{r\in \Omega }{\sum }C_{\mathrm{pac},k}(r;v_{\text{in}})].$$ (11)

The selected effective internal sound speed, $v_{\text{in}}^{*}$, was obtained by maximizing $J(v_{\text{in}})$. For mouse imaging, $v_{\text{in}}$ was exhaustively searched from 1510 to 1600  m/s in 1  m/s increments. The maximum ROI- and frame-averaged energy-ratio CF was obtained at $v_{\text{in}}^{*}=1563\;m/s$. This value is the discrete optimum of the production search and is treated as an effective correction parameter rather than a precise tissue sound-speed measurement.

### 2.3 Simulation Studies

Two main simulation studies were performed. The first was designed to isolate the mechanism by which phase aberration suppresses true signals in conventional CF. A hemispherical detector array with 1024 elements was generated using Fibonacci sampling, with a radius of 10 mm and the hemisphere located on the z≥0 side. The point source and ellipsoidal-prior center were deliberately offset, rather than concentrically aligned, to avoid an isotropic propagation condition and to create detector-dependent phase aberrations. The outer sound speed was 1490  m/s, and the inner effective speed was swept over 1490, 1540, 1600, 1660, 1720, 1780, and 1850  m/s.

Forward data were synthesized with a ray-based TOF model rather than a full wave-equation model. This choice was made to isolate the phase-coherence mechanism tested by PAC-CF. Each channel contained a Ricker-type pulse with a center frequency of 7.5 MHz, sampled at 80 MHz for 18  μs. Mild Gaussian noise with a channel SNR setting of 34 dB was added, and an inverse-distance geometrical spreading term was included. Reconstructions used a field of view of 7.0  mm×7.0  mm×4.0  mm centered at the source, with a voxel spacing of 0.10 mm. The red cross in the reconstruction panels marks the true source location.

The second simulation evaluated the robustness of PAC-CF to segmentation error in the ellipsoidal boundary. The detector array again contained 1024 Fibonacci-sampled elements on a 10-mm-radius hemisphere. The outer and inner sound speeds were 1490 and 1950  m/s, respectively. During reconstruction, only the boundary model used by PAC-CF was perturbed: translation error shifted the ellipsoid center along z by −15% to +15% of the true z semi-axis, and axis-length error scaled all three semi-axes by −15% to +15%. The received data were always generated from the true ellipsoid. For each perturbation, PAC-CF metrics were evaluated relative to the PAC-CF reconstruction obtained with the correct (0%-error) boundary.

To assess robustness beyond a matched ray-based propagation model, an additional two-dimensional nonmatched validation used k-wave full-wave forward data. The forward and reconstruction models differed in grid spacing, sound speeds, boundary shape, and frequency-response processing. Density heterogeneity and full-wave propagation were included in the forward model, whereas reconstruction retained the simplified straight-path two-region TOF. The complete protocol is provided in Supplementary Methods, Fig. S1, and Table S1 in the Supplementary Material.

### 2.4 Experimental Acquisition and Reconstruction

All experimental data were acquired using a custom-built hemispherical PACT system with a 1024-element ultrasound transducer array, 3.16-MHz center frequency, and 100-mm radius. Photoacoustic signals were excited by an Nd:YAG laser (Nimma-900, Beamtech Optronics Co, Ltd) operating at 1064 nm, with 100 mJ single-pulse energy and a 10-Hz repetition rate.

For the phantom experiment, two 100  μm diameter tungsten wires were crossed and immersed in 98% anhydrous ethanol contained by a barrel-shaped agar holder. Deionized water served as the external coupling medium. The water temperature measured during phantom imaging was 23.3°C, corresponding to $v_{\text{out}}=1492\;m/s$ after integer reporting. For conventional DAS and CF, a common homogeneous sound speed of 1447  m/s was empirically selected by exhaustively reconstructing the phantom data over a range of candidate sound speeds and comparing the resulting image quality. PAC-DAS and PAC-CF instead used $v_{\text{out}}=1492\;m/s$ and $v_{\text{in}}=1260\;m/s$ in the two-region model. The phantom quantitative analysis used one reconstructed frame. The ethanol-water speed mismatch produced a controlled heterogeneous acoustic path, allowing DAS, conventional CF, PAC-DAS, and PAC-CF to be compared under a known phase-aberration condition.

For in vivo imaging, a 10-week-old adult male nude mouse (NU/NU, Charles River Co) was used. The mouse was fasted for 24 h before imaging, body hair was removed with depilatory cream, and anesthesia was maintained with 1.5% vaporized isoflurane. The animal was fixed in a custom holder and immersed in a water bath during whole-body PACT. The measured water temperature was 21.4°C, corresponding to $v_{\text{out}}=1487\;m/s$ after integer reporting. The *in vivo* reconstruction and quantitative analysis used 20 correlation-selected frames from a single acquisition. No inter-acquisition repeatability assessment was performed; therefore, the *in vivo* experiment is presented as a single-animal proof-of-concept study. The animal protocol was approved by the Institutional Animal Care and Use Committee at Hangzhou Medical College.

### 2.5 Quantitative Metrics

Image quality was evaluated using true-source peak ratio, source CF, full width at half maximum (FWHM), peak localization error, signal retention, contrast-to-noise ratio (CNR), generalized contrast-to-noise ratio (gCNR), background fluctuation, relative peak amplitude, and line-profile comparisons. No signal-to-noise ratio (SNR)-based image-quality metric is reported in this manuscript. For phantom analysis, two fixed, method-independent sampling lines were positioned approximately perpendicular to the known wire trajectories, and the same sampling coordinates and ROI definitions were applied unchanged across DAS, conventional CF, PAC-DAS, and PAC-CF. The signal ROIs were band-shaped regions covering the two tungsten wires, and the background ROIs were selected from corner regions far from the wires. CNR was defined as

$$\mathrm{CNR}=\frac{\left|\text{mean}(\text{signal }\mathrm{ROI})-\text{mean}(\text{background }\mathrm{ROI})\right|}{\mathrm{std}(\text{background }\mathrm{ROI})}.$$ (12)

Background fluctuation was defined as the standard deviation of the background ROI. FWHM was calculated within local windows around the fixed sampling lines. For phantom comparison, signal retention and peak retention were normalized to the corresponding DAS signal-ROI mean and DAS wire peak, respectively. For boundary-error simulation, gCNR quantified the separability between the target ROI and background ROI distributions. The target and background histograms, $p_{t}(i)$ and $p_{b}(i)$, where i is the histogram-bin index, were constructed with 128 normalized bins over the joint intensity range, and gCNR was defined as

$$\mathrm{gCNR}=1-\overset{128}{\underset{i=1}{\sum }}\min(p_{t}\left(i\right),p_{b}(i)).$$ (13)

The heatmaps report the change in gCNR relative to the 0% boundary-error condition, not the absolute gCNR value

$$\Delta \mathrm{gCNR}=({\mathrm{gCNR}}_{0\%}-{\mathrm{gCNR}}_{\text{error}})\times 100\%.$$ (14)

Here, ${\mathrm{gCNR}}_{0\%}$ and ${\mathrm{gCNR}}_{\text{error}}$ denote the zero-boundary-error and perturbed-boundary gCNR values, respectively. Thus, a negative ΔgCNR indicates that the perturbed-boundary result has a higher gCNR than the 0% boundary-error result. The true-source peak ratio used in the principle simulation was defined as

$$R_{\text{source}}=\frac{I(r_{s})}{\underset{r}{\max}\;I(r)},$$ (15)

where I(rs) is the reconstructed value at the true source location rs and maxr I(r) is the maximum value over all voxels in the same reconstructed image. This metric indicates whether the true source remains the image peak; it is not a physical initial-pressure ratio. In the boundary-error simulation, PAC-CF signal retention was based on the mean target-ROI intensity

$$S_{\text{retention}}=\frac{\mu_{t,\text{error}}}{\mu_{t,0\%}}.$$ (16)

In Eq. (16), $\mu_{t,\text{error}}$ and $\mu_{t,0\%}$ are the mean target-ROI intensities for the perturbed-boundary and zero-boundary-error PAC-CF reconstructions, respectively. Signal loss was then computed as $(1\text{-}S_{\text{retention}})\times 100\%$. For *in vivo* line-profile analysis, relative peak amplitude was defined as the ratio between the vessel-peak intensity of a method and the corresponding DAS peak at the same fixed vessel location. Because coherence weighting changes the absolute image-amplitude scale, this metric is interpreted together with PAC-CF-to-CF improvement, CNR, and background fluctuation. Background fluctuation was calculated as the standard deviation of the normalized background profile after excluding vessel-peak neighborhoods. Because the exact profile lines and representative vessel positions were selected retrospectively within the predefined DAS-based ROIs, these metrics are interpreted as descriptive, mechanism-oriented comparisons rather than blinded global performance estimates.

The computationally intensive backprojection and PAC-CF reconstruction steps were implemented on an NVIDIA GPU using CUDA. In the supplied implementation, a single-frame reconstruction of a 500×500×500  voxel volume required 2.39 s on an NVIDIA RTX 4090 GPU using CUDA version 12.4. The simulation code, reconstruction code, and data required for reconstruction are available at https://github.com/GaoXiali/Phase-Aberration-Corrected-Coherence-Factor-Beamforming.

## 3 Results

### 3.1 PAC-CF Prevents Phase-Aberration-Induced Suppression of a True Source

Figure 2 presents the main principle simulation. Figures 2(a1) and 2(a2) show the detector array, point source, and ellipsoidal prior in three-dimensional and cross-sectional views. The central reconstruction block compares DAS, conventional CF, PAC-DAS, and PAC-CF in independently normalized linear displays at representative sound-speed differences; the red cross marks the true source location in each image. When the sound-speed difference was zero, all methods localized the peak at the true source and the true-source peak ratio was 1. As the sound-speed difference increased, homogeneous-TOF compensation became increasingly inaccurate. At $v_{\text{in}}=1660\;m/s$, corresponding to a 170  m/s sound-speed difference, the standard deviation of the homogeneous-TOF error was ∼154.6  ns and the error range was ∼597.0  ns. At $v_{\text{in}}=1850\;m/s$, corresponding to a 360  m/s sound-speed difference, these values increased to ∼293.8  ns and 1134.3 ns, respectively.

**Fig. 2:**
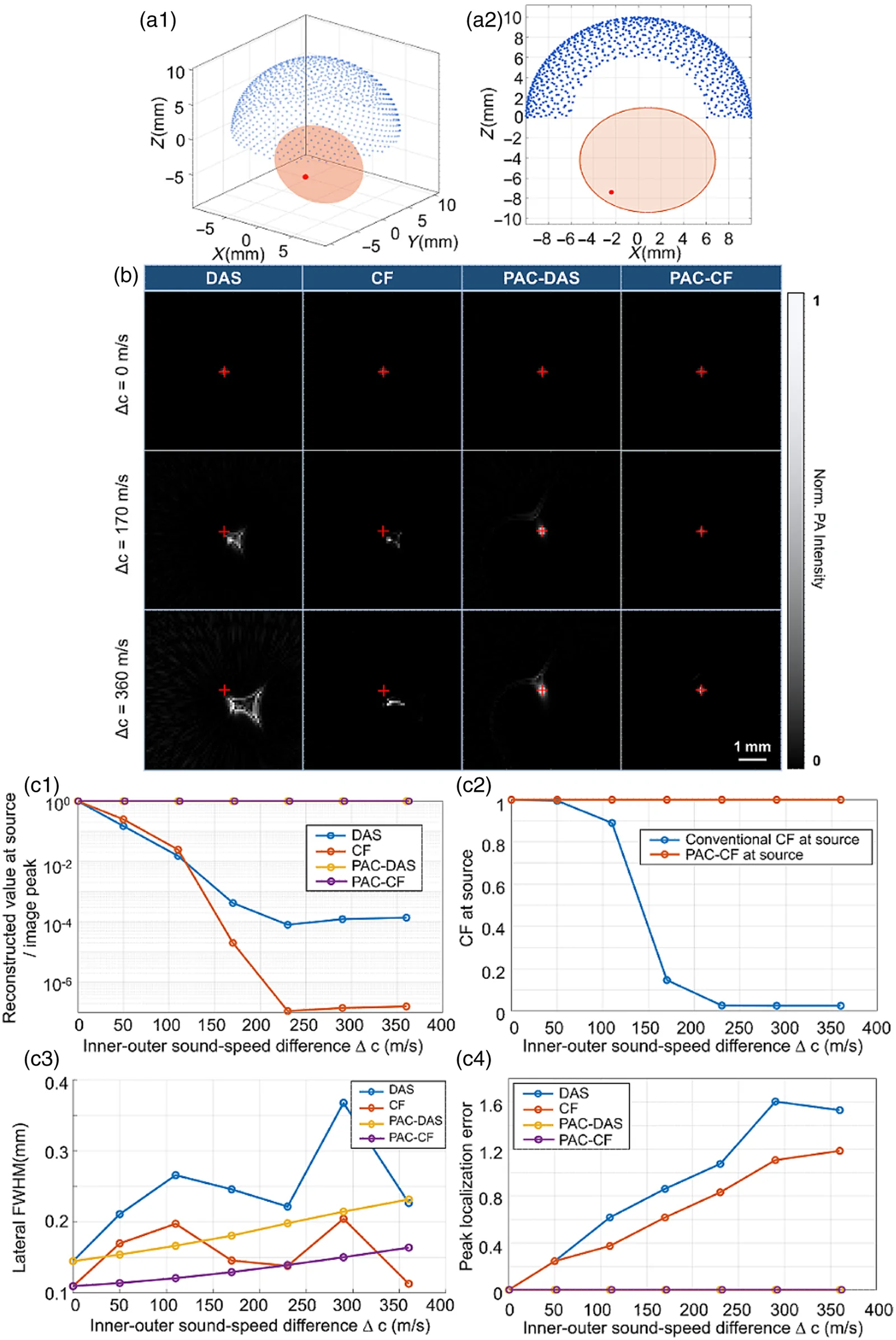
Principle simulation showing phase-aberration-induced suppression by conventional CF and recovery by PAC-CF. (a1) Three-dimensional geometry of the hemispherical detector array, ellipsoidal two-region prior, and point source. (a2) Cross-sectional geometry. (b) DAS, conventional CF, PAC-DAS, and PAC-CF reconstructions at sound-speed differences of 0, 170, and 360  m/s; the red cross marks the true source. Each reconstruction subimage is independently normalized to its own maximum intensity and displayed on a linear scale from 0 to 1, as indicated by the shared Min-Max colorbar. (c1)–(c4) Source-to-peak ratio, source energy-ratio CF, lateral FWHM, and localization error across the complete sweep. PAC-DAS shows the DAS reconstruction after two-region TOF correction, whereas PAC-CF shows the reconstruction using the phase-corrected coherence factor. Conventional CF progressively suppresses and mislocalizes the true source under homogeneous-delay mismatch, whereas PAC-CF preserves coherence and localization after two-region TOF correction.

The quantitative plots in Fig. 2 show the consequence of this delay error for CF weighting. The true-source peak-ratio plot shows progressive suppression of the real source by conventional CF, whereas PAC-CF retains the true source as the dominant peak. The source-CF plot shows that the homogeneous-delay source CF decreased to 0.00794 at a 170  m/s sound-speed difference and to ∼0.00039 to 0.00042 at sound-speed differences of 230  m/s or higher. The FWHM and localization-error plots show that suppression was accompanied by loss of spatial reliability. In the most severe tested condition, $v_{\text{in}}=1850\;m/s$, the true-source peak ratio of conventional CF was ∼1.6e-7 and the peak localization error was ∼1.18  mm. Using the corrected two-region TOF, both PAC-DAS and PAC-CF maintained the true source as the image peak and kept the localization error at 0 across all tested sound-speed differences. PAC-DAS therefore isolates the contribution of delay correction without final CF weighting, whereas PAC-CF further applies the conventional energy-ratio CF, preserves the source CF at ∼0.983 to 0.984 across the sweep, and yields a narrower lateral response than PAC-DAS.

### 3.2 PAC-CF Tolerates Moderate Boundary-Prior Error

Figure 3 evaluates whether PAC-CF requires a perfect ellipsoidal boundary. Figure 3(a1) shows the true simulation geometry. Figure 3(a2) illustrates translation perturbation of the boundary model, and Fig. 3(a3) illustrates uniform scaling of the ellipsoid axes. In all cases, the raw channel data were synthesized using the true ellipsoid; only the boundary model used in PAC-CF reconstruction was perturbed.

**Fig. 3:**
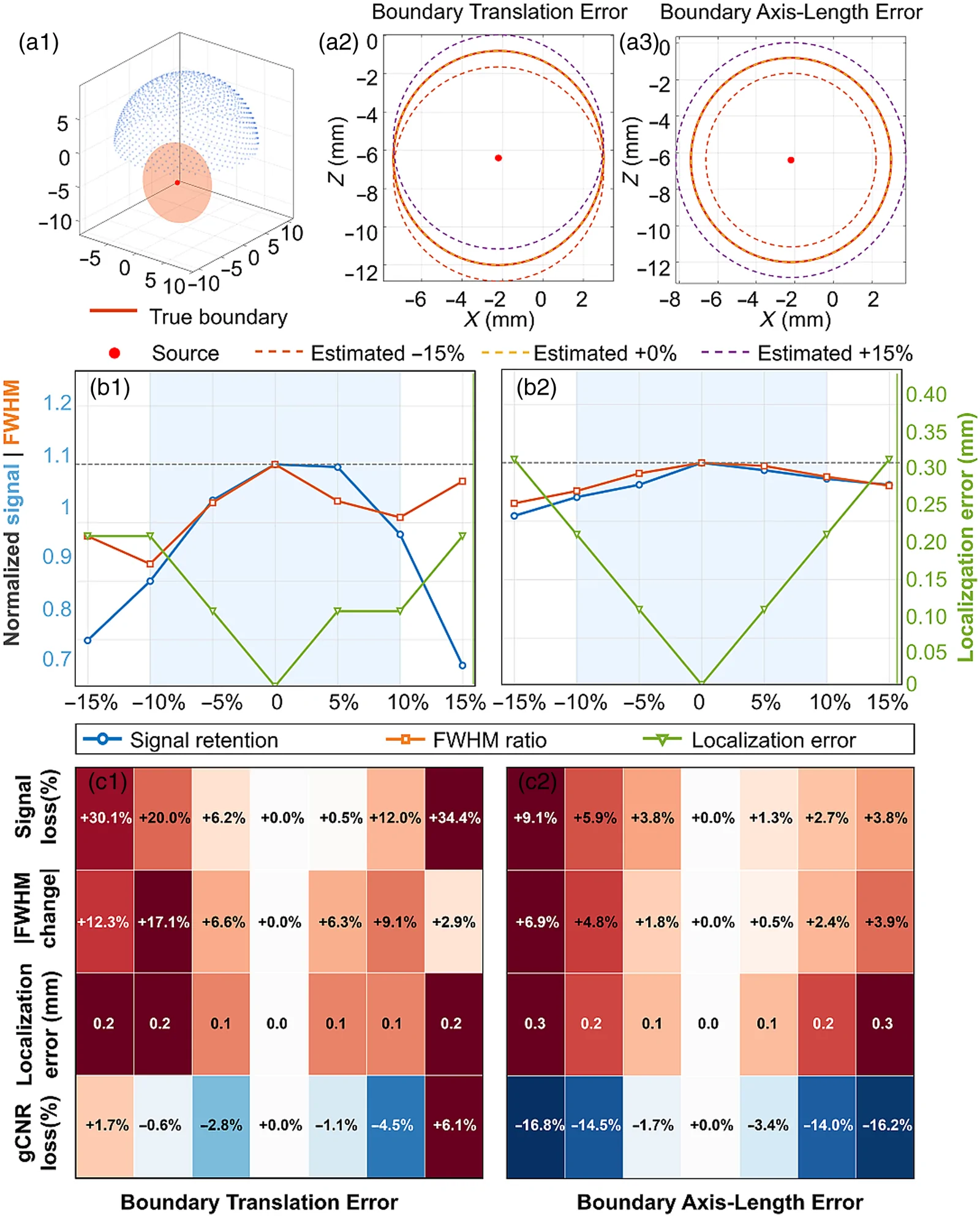
Robustness of PAC-CF to boundary-model error. (a1) True geometry of the detector array, ellipsoid, and point source. (a2) Translation-error model, in which the ellipsoid center is shifted along z from −15% to +15% of the true z semi-axis. (a3) Axis-length-error model, in which all semi-axes are scaled from −15% to +15%. (b1), (b2) PAC-CF signal retention, FWHM ratio, and localization error under translation and axis-length errors, respectively; the shaded region marks the ±10% range. Signal retention and FWHM ratio are normalized to the 0%-error PAC-CF reconstruction. (c1), (c2) Heatmaps of signal loss, absolute FWHM change, localization error, and gCNR loss relative to the 0%-error PAC-CF reconstruction. Negative gCNR-loss values indicate a higher gCNR than the zero-error reference.

The line plots in Figs. 3(b1) and 3(b2) summarize PAC-CF signal retention, FWHM ratio, and localization error under translation and axis-length errors. Across the full −15% to +15% range, translation errors yielded signal retention of 0.656 to 1.000, an FWHM ratio of 0.829 to 1.000, and localization error no larger than 0.2 mm. Axis-length errors yielded signal retention of 0.909 to 1.000, an FWHM ratio of 0.931 to 1.000, and localization error no larger than 0.3 mm. Within the shaded ±10% range, signal retention remained at least 0.800 for translation errors and 0.941 for axis-length errors. The heatmaps in Figs. 3(c1) and 3(c2) report the same results as signal loss, absolute FWHM change, localization error, and gCNR loss relative to the zero-error PAC-CF reconstruction. The largest signal loss, 34.4%, occurred at the extreme +15% translation error, whereas the largest FWHM change was 17.1% at −10% translation error. The gCNR-loss values were nonmonotonic: for translation errors they ranged from −4.5% to +6.1%, whereas every nonzero axis-length perturbation yielded a negative value, ranging from −16.8% to −1.7%. A negative gCNR-loss value indicates that the perturbed reconstruction had a higher gCNR than the zero-error reference. These results support robustness to moderate boundary mismatch, rather than unlimited tolerance to segmentation error.

In the supplementary nonmatched full-wave simulation, PAC-DAS and PAC-CF reduced the peak localization error from 0.582 mm for DAS and 0.506 mm for conventional CF to 0.113 mm, while retaining true-source-to-peak ratios of 0.946 and 0.936, respectively. The corresponding ratios were 0.229 for DAS and 0.055 for conventional CF. PAC-CF further reduced the normalized background standard deviation relative to PAC-DAS. The complete nonmatched full-wave validation is provided in the Supplementary Material, with the simulation configuration and reconstruction images in Fig. S1 and the quantitative comparison in Table S1.

### 3.3 Crossed-Wire Phantom Validation under Phase Mismatch

The crossed-wire phantom experiment tested whether PAC-CF can recover target information that conventional CF suppresses under a controlled practical mismatch between ethanol and water. Figures 4(a1) and 4(a2) show the schematic and photograph of the phantom. Two 100 μm tungsten wires were immersed in anhydrous ethanol within the agar holder, whereas water served as the coupling medium. PAC-DAS and PAC-CF used the measured water speed $v_{\text{out}}=1492\;m/s$ and the ethanol-region speed $v_{\mathrm{in}}=1260\;m/s$. This configuration caused the wire-generated PA signals to accumulate detector-dependent phase aberrations before detection.

**Fig. 4:**
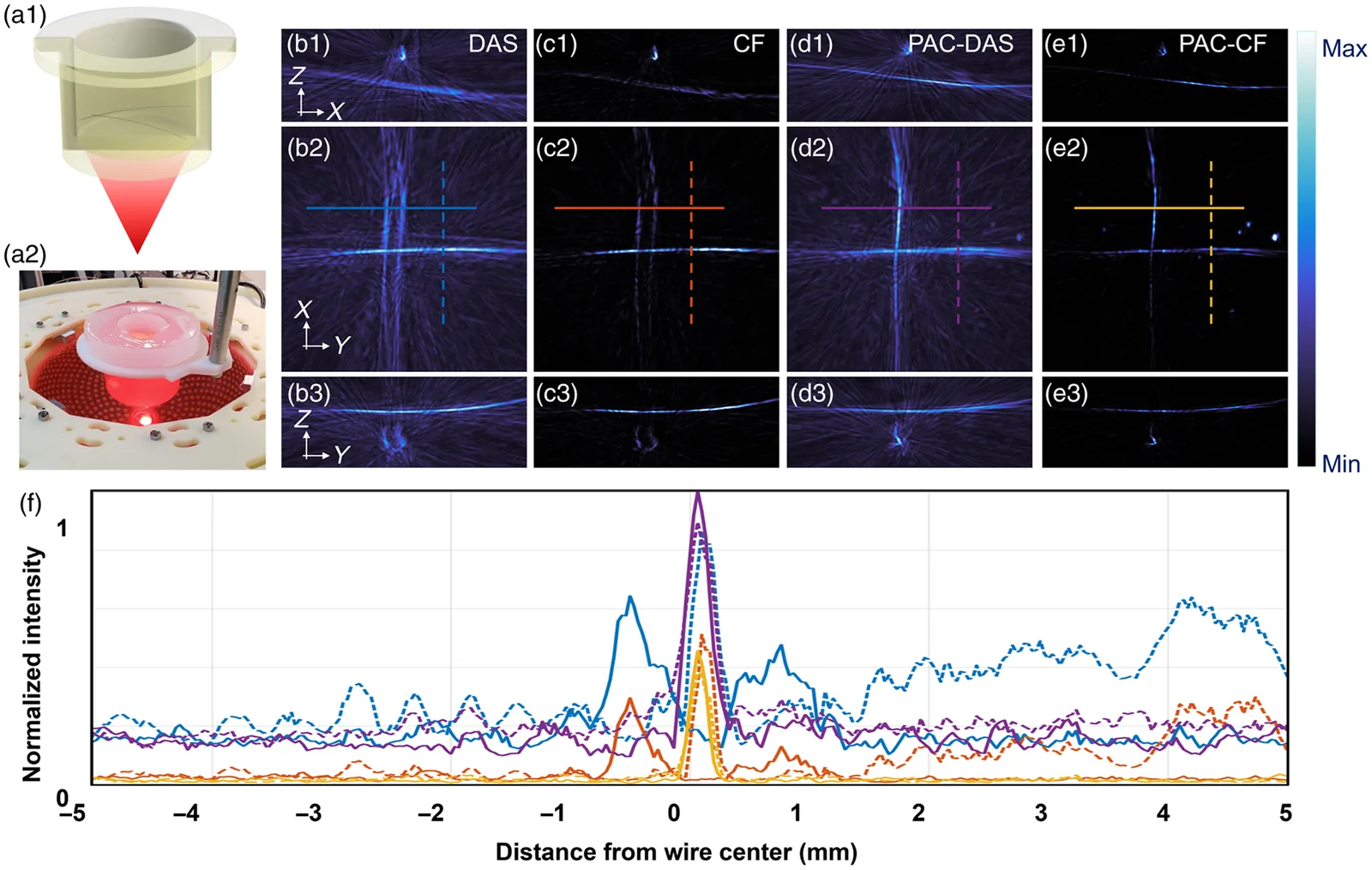
Phantom validation of PAC-CF under phase mismatch. (a1), (a2) Schematic and photograph of the crossed-100  μm tungsten-wire phantom in an ethanol-filled region surrounded by water. (b1)–(b3) DAS projections in XZ, XY, and ZY views. (c1)–(c3) Conventional CF projections. (d1)–(d3) PAC-DAS projections after two-region TOF correction without final CF weighting. (e1)–(e3) PAC-CF projections using two-region TOF correction followed by conventional energy-ratio CF weighting. (f) Four-method normalized intensity profiles extracted from the indicated sampling lines. PAC-DAS isolates the effect of the corrected delay, whereas PAC-CF demonstrates the combined effect of corrected phase alignment and final CF weighting. The two sampling lines were positioned approximately perpendicular to the known wire trajectories and applied unchanged to all four methods.

Figures 4(b1)–4(b3) show DAS reconstructions in the XZ, XY, and ZY projections. DAS partially recovered the wires but exhibited strong sidelobes and streak-like background artifacts; in the XY projection, one wire appeared split and spatially displaced. Figures 4(c1)–4(c3) show conventional CF, which reduced part of the incoherent background but also suppressed wire signals whose apparent coherence was degraded by the single-speed delay model. Figures 4(d1)–4(d3) show PAC-DAS, which applied the two-region TOF correction without final CF weighting. The corrected delay recovered stronger and more concentrated wire responses, but more off-target fluctuation remained than in PAC-CF. Figures 4(e1)–4(e3) show PAC-CF. By applying the conventional energy-ratio CF to the phase-corrected channel contributions, PAC-CF combined the structural recovery provided by the corrected delay with further suppression of incoherent background. The normalized line profiles in Fig. 4(f), extracted along the fixed sampling lines shown in the XY projections, support this combined effect. Because the relative image amplitudes differed substantially among the four methods, method-dependent linear display ranges were used to maintain visibility of the reconstructed structures; this display scaling was not used for the line profiles or quantitative calculations.

The quantitative phantom metrics further show why both components are required for the overall PAC-CF performance. The mean FWHM values for DAS, conventional CF, PAC-DAS, and PAC-CF were 0.400, 0.250, 0.283, and 0.217 mm, respectively, whereas the corresponding background fluctuations were 0.0231, 0.0063, 0.0191, and 0.0031 and the CNR values were 8.55, 11.75, 13.43, and 18.20. PAC-DAS increased the signal retention and mean peak retention relative to DAS to 1.196 and 1.256, respectively, confirming that two-region TOF correction alone recovered and refocused wire signal. After final CF weighting, PAC-CF yielded a signal retention of 0.237 and a mean peak retention of 0.555; the latter remained slightly higher than the 0.535 obtained with conventional CF, whereas PAC-CF achieved the lowest background fluctuation, narrowest mean FWHM, and highest CNR of the four methods. Thus, PAC-DAS serves as a mechanism ablation showing that the PAC-CF improvement cannot be explained by delay correction alone: the corrected delay restores the phase alignment on which CF operates, and their combination provides the strongest balance between target concentration and background suppression.

### 3.4 *In Vivo* Mouse Vascular Imaging

Figure 5 evaluates PAC-CF in mouse PACT data. Figure 5(a) shows a whole-mouse maximum-intensity projection with two representative ROIs: the liver region marked by the blue dashed box and the mesenteric artery region marked by the yellow dashed box. The liver profile was extracted from the XY view at y=3.30  mm, and the mesenteric artery profile was extracted from the XY view at y=−7.60  mm. The same spatial positions were used for DAS, conventional CF, PAC-DAS, and PAC-CF. PAC-CF used $v_{\text{out}}=1487\;m/s$ from the measured water temperature and $v_{\text{in}}=1563\;m/s$ selected by the coherence-based search. These broad ROIs were selected from the initial homogeneous-delay DAS volume before PAC-DAS or PAC-CF images were viewed because they were relatively deep, feature-rich regions in which heterogeneous acoustic paths are expected to have a stronger effect. The exact profile lines and representative vessel positions were subsequently selected within these predefined ROIs for retrospective, mechanism-oriented comparison and then applied unchanged to all methods. A single optimized internal speed was used for both regions; the liver ROI was spatially separate from the speed-optimization ROI, whereas the mesenteric artery ROI overlapped it.

**Fig. 5:**
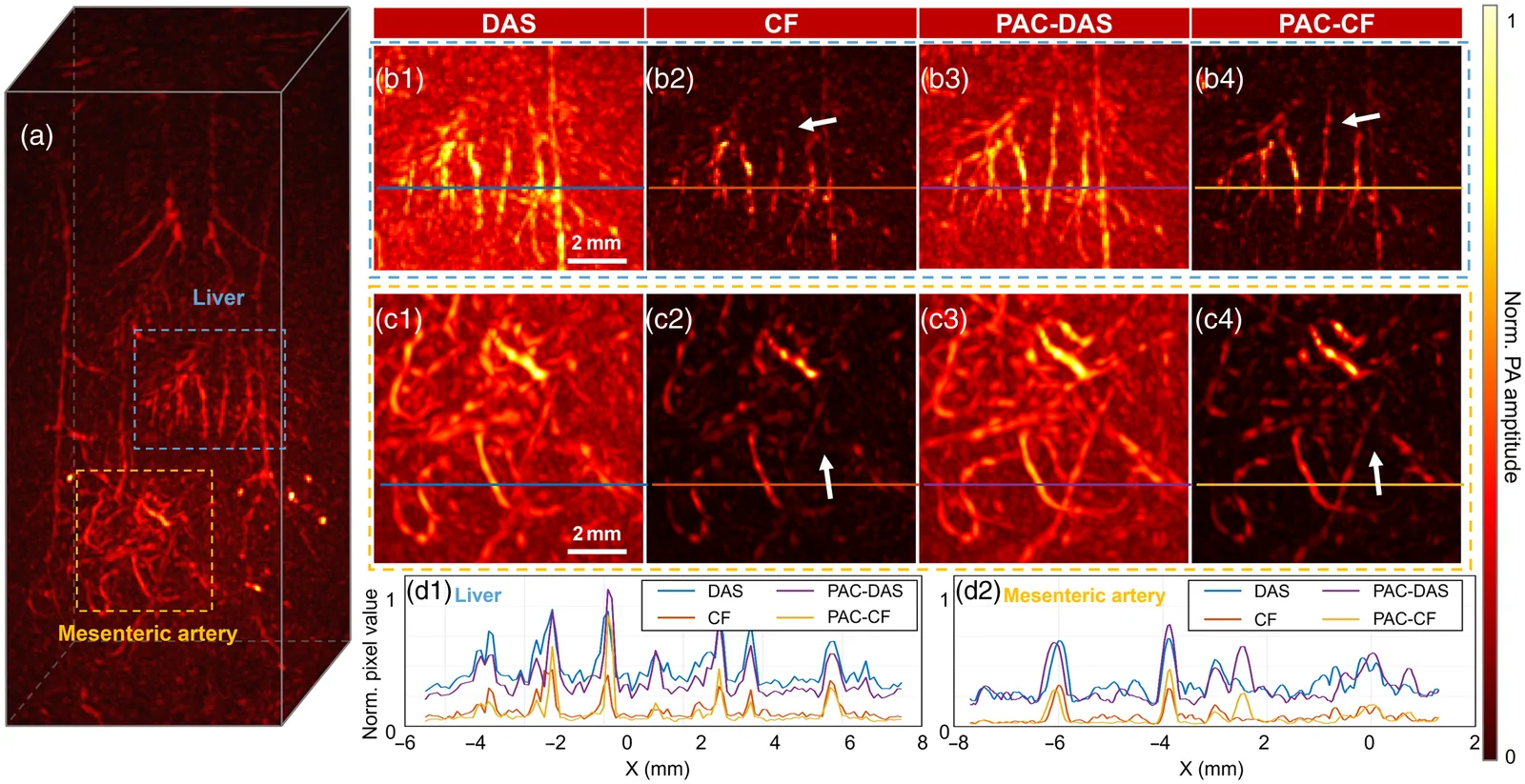
Preservation and enhancement of vascular features by PAC-CF in mouse PACT reconstruction. (a) Whole-mouse maximum-intensity projection with the liver and mesenteric artery ROIs marked by blue and yellow dashed boxes, respectively. (b1)–(b4) Liver-region reconstructions using DAS, conventional CF, PAC-DAS, and PAC-CF. (c1)–(c4) Mesenteric-artery-region reconstructions using the same four methods. (d1), (d2) Four-method normalized intensity profiles extracted at the identical horizontal sampling locations indicated in the corresponding reconstruction panels. PAC-DAS isolates the contribution of the corrected two-region delay, whereas PAC-CF additionally applies the energy-ratio coherence weight to suppress background interference. Each reconstruction panel was normalized to its own maximum for visualization; quantitative relative peak amplitudes were calculated from the raw projection values. White arrows indicate representative weak vascular features attenuated by conventional CF and more clearly recovered by PAC-CF. Scale bars: 2 mm.

A separate sensitivity analysis of 33 ROI configurations produced a nominal discrete maximum of 1560  m/s, close to the 1563-m/s production-search result; the perturbed-ROI maxima remained relatively close within 1557.5 to 1580.0  m/s, with none at a search boundary. The curves formed a broad high-coherence plateau rather than a sharply identifiable optimum; the complete protocol and curves are provided in Fig. S2 in the Supplementary Material.

Figures 5(b1)–5(b4) compare DAS, conventional CF, PAC-DAS, and PAC-CF in the liver ROI. DAS preserved abundant vascular signals but also retained elevated background and sidelobe artifacts. Conventional CF produced a cleaner background but attenuated the arrowed weak vascular feature. PAC-DAS used the corrected two-region delay without coherence weighting and improved vascular continuity while retaining a DAS-like background. PAC-CF combined the corrected delay with energy-ratio CF weighting, preserving the recovered feature while further suppressing background interference. Figures 5(c1)–5(c4) show the mesenteric artery ROI. The same pattern was observed: PAC-DAS improved continuity after delay correction but retained more background, whereas PAC-CF preserved fine vascular morphology with stronger background suppression than PAC-DAS. The profiles in Figs. 5(d1) and 5(d2) were extracted at the identical horizontal sampling locations indicated in panels (b1)–(b4) and (c1)–(c4), respectively. The dominant vessel peaks remain spatially consistent among the four methods, indicating recovery of existing structures rather than artificial spatial displacement.

Quantitative line-profile analysis further supports this interpretation. The reported *in vivo* peak values are relative peak amplitudes normalized to the corresponding DAS vessel peak at the same spatial location; because CF weighting changes absolute image scaling, the most relevant comparison is between PAC-CF and conventional CF together with CNR and background fluctuation. In the liver ROI, PAC-DAS increased the average relative peak amplitude to 1.234, whereas conventional CF and PAC-CF produced values of 0.0081 and 0.0151, respectively; thus, PAC-CF increased the retained peak amplitude by 87.4% relative to conventional CF. The background fluctuations for DAS, conventional CF, PAC-DAS, and PAC-CF were 0.0787, 0.0362, 0.0642, and 0.0252, respectively, and the corresponding CNR values were 4.23, 5.35, 6.02, and 12.53. In the mesenteric artery ROI, PAC-DAS yielded an average relative peak amplitude of 1.235, whereas conventional CF and PAC-CF yielded 0.0104 and 0.0174, respectively, corresponding to a 67.0% improvement of PAC-CF over conventional CF. The background fluctuations for the four methods were 0.0433, 0.0198, 0.0522, and 0.0163, and the corresponding CNR values were 5.32, 6.17, 5.88, and 11.00. These results separate the two contributions: delay correction in PAC-DAS recovered vessel-peak amplitude and continuity, whereas subsequent energy-ratio CF weighting in PAC-CF provided the strongest background suppression and CNR.

## 4 Discussion and Conclusion

The results demonstrate a specific limitation of conventional CF in hemispherical PACT. CF is often interpreted as an artifact-suppression weight, but its decision depends on the delay model used before the factor is calculated. When this model is wrong, a real absorber can be assigned a low coherence value. In the principle simulation, increasing sound-speed difference reduced the apparent source CF and caused conventional CF to suppress and mislocalize the true source. PAC-CF applied the two-region TOF correction before coherence estimation and restored the source as a high-coherence structure.

The methodological contribution of PAC-CF lies in extending an ellipsoidal two-region TOF correction from the coherent backprojection term to the angle-weighted channel contributions evaluated by the final conventional CF. This distinction is important because propagation-model mismatch affects not only DAS focusing but also the validity of the coherence decision: a true absorber can receive an artificially low CF weight when its detector-dependent delays are not adequately compensated. PAC-CF therefore uses partial TOF correction to improve both coherent reconstruction and the reliability of the subsequent CF weighting.

This formulation integrates elements that prior studies demonstrated in different settings: CF-guided homogeneous effective-speed selection with final CF weighting in two-dimensional PA beamforming;^21^ US-coherence-based selection of mean or dual-region sound speeds in full-ring USPACT;^22^^,^^23^ and ellipsoidal dual-speed correction in three-dimensional hemispherical UBP reconstruction.^24^^,^^25^ In PAC-CF, coherence has two connected roles: selecting the ROI-optimized effective internal sound speed from PA data and weighting the final image after partial phase alignment. The contribution therefore lies in this PA-only three-dimensional integration rather than in the ellipsoidal model, coherence-based speed search, or conventional CF definition individually.

The boundary-error simulation suggests that PAC-CF does not require exact segmentation. This is encouraging because practical tissue boundaries are difficult to define precisely from PA data alone. Within ±10% perturbations, PAC-CF retained at least 0.800 of the zero-error target signal for translation errors and at least 0.941 for axis-length errors, with localization errors no larger than 0.2 mm. The reduction to 0.656 signal retention at the extreme +15% translation error also shows that the tolerance is not unlimited. Thus, the prior should capture the dominant phase-aberrating region, but moderate boundary mismatch can be tolerated.

The phantom and *in vivo* experiments further support PAC-CF as a combined correction-and-weighting strategy. In the crossed-wire phantom, PAC-DAS confirmed that two-region TOF correction alone recovered and refocused wire responses, but it retained more background fluctuation than PAC-CF. PAC-CF applied the conventional energy-ratio CF after this partial phase alignment and achieved the narrowest mean FWHM, lowest background fluctuation, and highest CNR among the four methods. The PAC-DAS ablation therefore shows that the observed PAC-CF benefit is not attributable solely to delay correction; it arises from combining improved phase alignment with the subsequent coherence decision. In mouse data, the same relationship was observed: PAC-DAS improved vessel-peak retention and structural continuity but retained a relatively high background, whereas PAC-CF preserved the recovered features while providing the additional background suppression associated with energy-ratio coherence weighting. The line-profile alignment in Fig. 5 indicates that the enhanced features were spatially consistent with structures present in DAS, conventional CF, or PAC-DAS.

Several limitations should be noted. First, the current implementation uses a simplified ellipsoidal two-region prior. Real tissue contains spatially varying sound speed, attenuation, density variation, and possible refractive effects at curved interfaces. PAC-CF should therefore be understood as a phase-aberration reduction strategy, not a complete acoustic inverse solution. Second, the current parameter search is scalar and ROI-based; more complex anatomy may require multiple regions or stronger priors. Third, the main simulations used ray-based forward data to isolate the coherence mechanism. A supplementary nonmatched k-wave simulation provides full-wave forward validation, but reconstruction still uses the simplified straight-path two-region model; incorporating refraction, diffraction, and spatially varying acoustic properties into the inverse model remains future work. The released code and reconstruction data support reproducibility, but independent tests on additional systems and samples will still be needed to define the practical operating range of the framework. The ROI-sensitivity analysis showed that the resulting speeds were relatively close but that the discrete maximum varied within the broad objective plateau; thus, the selected value should not be interpreted as a high-precision estimate. Detailed ROI perturbations and objective curves are reported in Fig. S2 in the Supplementary Material. The single-animal *in vivo* experiment did not include an inter-acquisition repeatability assessment; its retrospective, region-specific profile analysis is therefore interpreted as descriptive proof-of-concept evidence rather than blinded or global validation.

The compact ellipsoidal prior should also be interpreted as one realization of the PAC-CF framework rather than a fixed requirement. In more complex biological or clinical scenarios, such as human breast imaging, the true tissue boundary and sound-speed distribution can be irregular and spatially heterogeneous. PAC-CF could therefore be extended by replacing the ellipsoidal prior with non-ellipsoidal anatomical masks derived from an initial DAS reconstruction, co-registered ultrasound, or other multimodal structural information. Such masks could provide voxel-dependent path partitioning or region-wise effective sound speeds while preserving the same central idea: the TOF model is corrected before CF is evaluated. This extension may improve generalization in anatomically complex samples without changing the coherence-weighting principle of PAC-CF.

The proposed method should be viewed as a flexible framework for partial phase-aberration correction rather than a closed endpoint. More advanced acoustic priors, automatic region definition, or measured sound-speed maps could be incorporated to improve the delay estimate before CF is evaluated. Similarly, PAC-CF can be paired with modified CF, lag-coherence, or other spatial-coherence measures after phase alignment. The essential principle is that coherence weighting should be applied only after the delay model has made the true signal as coherent as practically possible.

PAC-CF extends an ellipsoidal two-region TOF correction to the angle-weighted channel contributions used for final conventional energy-ratio CF evaluation in three-dimensional hemispherical PA-only PACT. By correcting the TOF used before the conventional energy-ratio CF is evaluated, PAC-CF reduces phase-aberration-induced over-suppression. Simulations, phantom data, and *in vivo* mouse reconstructions show that PAC-CF preserves the artifact-suppression advantage of CF, whereas recovering structures that conventional CF can attenuate. The method provides a practical route toward more reliable coherence-weighted PACT reconstruction.

## Supplementary Material

10.1117/1.JBO.31.10.106003.s01

## Data Availability

To facilitate reproducibility, the released implementation documents the complete three-dimensional workflow, including ellipsoidal path partitioning, angular weighting, multiframe sound-speed optimization, and final PAC-CF reconstruction. The simulation code, reconstruction code, data required for reconstruction, and raw analysis data for the quantitative metrics are available at https://github.com/GaoXiali/Phase-Aberration-Corrected-Coherence-Factor-Beamforming.
