# Supplementary material for: Phase-aberration-corrected coherence factor beamforming for photoacoustic computed tomography
Source: J Biomed Opt. 2026 Oct 9;31(10):106003. doi: 10.1117/1.JBO.31.10.106003 (PMC13652113; doi:10.1117/1.JBO.31.10.106003)
Supplement: Supplementary file 1 [file JBO_031_106003_SD001.docx]

**Supplementary Material**

**Phase-Aberration-Corrected Coherence Factor Beamforming for Photoacoustic Computed Tomography**

**Supplementary Methods 1.** Non-matched full-wave k-Wave validation

To reduce model-matching bias, photoacoustic sensor data were generated with the two-dimensional k-Wave solver and reconstructed using a deliberately different straight-path model. The full-wave forward grid contained 288 x 288 points with 0.10-mm spacing, a 20-point PML, and a CFL number of 0.20. The external and internal sound speeds were 1480 and 1560 m/s, and the corresponding densities were 1000 and 1050 kg/m3. The true tissue region was based on an ellipse with semi-axes of 5.50 and 6.50 mm centered at (0, -2.00) mm, with normalized boundary perturbations 0.060 cos(3theta) and 0.030 sin(5theta). The point source was centered at (-1.8, -5.0) mm with a radius of 0.20 mm. A 240-element sensor arc of 11-mm radius covered 20-160 degrees. The simulated detector response had a 3.16-MHz center frequency and 80% bandwidth, and Gaussian noise was added at 35 dB SNR.

Reconstruction used a separate 188 x 139 grid with 0.08-mm spacing. The assumed external and internal sound speeds were 1482 and 1563 m/s. The received data were additionally processed with a deliberately different 3.00-MHz, 70% Gaussian bandpass; this step represents preprocessing mismatch rather than inversion of the detector response. The reconstruction boundary was a smooth ellipse with semi-axes of 5.35 and 6.40 mm centered at (0.15, -1.85) mm. Reconstruction used straight-path two-region TOFs and did not model refraction, diffraction, density, or attenuation. DAS, conventional CF, PAC-DAS, and PAC-CF used the same received data and unit channel weights. Both CF variants used the conventional energy-ratio definition.

**Supplementary Results 1.**

Under the non-matched condition, the true-source-to-peak ratios were 0.229 for DAS, 0.055 for conventional CF, 0.946 for PAC-DAS, and 0.936 for PAC-CF. Peak localization errors were 0.582, 0.506, 0.113, and 0.113 mm, respectively. Thus, the two-region delay correction substantially improved source retention and localization despite the forward-reconstruction mismatch. Relative to PAC-DAS, PAC-CF reduced the x-direction FWHM from 0.56 to 0.40 mm and reduced the peak-normalized background standard deviation from 3.878 x 10^-3 to 2.665 x 10^-5. The gCNR values do not rank all methods identically; PAC-CF exceeded conventional CF but was lower than DAS and PAC-DAS. Accordingly, this validation is interpreted as evidence of improved localization, true-source retention, and incremental background suppression rather than superiority in every metric.

**Fig. S1.** Non-matched full-wave validation. (a) Heterogeneous k-Wave forward medium, sensor arc, source, and the smooth ellipse assumed during reconstruction. The actual forward boundary contains harmonic perturbations and does not coincide with the assumed ellipse. (b) DAS, conventional CF, PAC-DAS, and PAC-CF reconstructions shown on linear and 40-dB scales. Display ranges are identical within each row, and the cyan cross marks the true source location.

**Table S1.** Quantitative comparison in the non-matched full-wave simulation.

| **Method** | **Source/ peak** | **Peak error (mm)** | **FWHM x/y (mm)** | **CNR** | **gCNR** | **Background SD** |
| --- | --- | --- | --- | --- | --- | --- |
| DAS | 0.2289 | 0.5824 | 0.48/1.28 | 68.85 | 0.9387 | 3.013 x 10^-3 |
| CF | 0.0548 | 0.5060 | 0.24/0.64 | 4135.97 | 0.5670 | 2.864 x 10^-5 |
| PAC-DAS | 0.9464 | 0.1131 | 0.56/0.24 | 93.96 | 0.9231 | 3.878 x 10^-3 |
| PAC-CF | 0.9359 | 0.1131 | 0.40/0.24 | 9084.81 | 0.7320 | 2.665 x 10^-5 |

Note: Each image was normalized by its own peak before image-domain metrics were computed. The large CNR values for CF-weighted images reflect near-zero background variance and should be interpreted together with source retention, localization, gCNR, and background standard deviation.

**Supplementary Methods 2.** ROI sensitivity of the PA-only sound-speed objective

A nominal cuboidal ROI centered at (-3.2, -6.1, 0) mm with extents of 9 x 9 x 30 mm was used. Before defining or evaluating any perturbed ROI, the same 20 frames (indices 11, 12, 13, 20-34, 44, and 45) were fixed for all candidates. The assumed internal sound speed was evaluated from 1510 to 1600 m/s in 2.5-m/s increments. ROI size was varied isotropically by +/-5%, +/-10%, +/-15%, and +/-20%. Separately, the ROI center was shifted along x, y, and z by the same percentages of the corresponding nominal half-extent; the largest shifts were 0.9, 0.9, and 3.0 mm, respectively. The sensitivity analysis therefore contained 33 unique ROI configurations. For computational tractability, the ROI grid density was 5 points/mm. For each ROI and candidate speed, the conventional energy-ratio CF was averaged over ROI voxels within each frame and then averaged over the 20 fixed frames. This reduced sensitivity protocol was distinct from the production search, which used 1-m/s increments, and 15 grid points/mm.

**Supplementary Results 2.**

The nominal ROI produced a discrete maximum at 1560 m/s, close to the 1563-m/s value selected by the production 1-m/s exhaustive search. Because the sensitivity grid used 2.5-m/s increments, 1563 m/s was not a candidate; the closest candidate was 1562.5 m/s. Across the 32 perturbed ROIs, the maximizing speeds ranged from 1557.5 to 1580.0 m/s, all within 17 m/s of the production-search value, and none occurred at the 1510- or 1600-m/s search boundaries. The objective curves were shallow: the relative full-range variation, defined as (maximum - minimum)/maximum x 100%, ranged from 0.121901% to 0.319057% with a median of 0.261659%. For the nominal ROI, the objective values at 1560 and 1562.5 m/s differed by 7.8074 x 10^-8, corresponding to only 0.007954% of the peak. Thus, the individual discrete maxima vary with local ROI perturbations, but the selected speeds remain relatively close within a broad interior high-coherence plateau. These data support interpreting the selected speed as an ROI-optimized effective correction parameter rather than a high-precision tissue sound-speed measurement.

**Fig. S2.** Sensitivity of the PA-only coherence-based sound-speed objective to ROI initialization. The nominal ROI was subjected to isotropic size changes and independent shifts along x, y, and z from +/-5% to +/-20%. Each curve was normalized by its own maximum for visualization only; the unnormalized energy-ratio CF values were used for optimization and quantitative analysis. Crosses indicate the discrete maximizing speed for each ROI, and the vertical dotted line denotes the 1563-m/s value selected by the production 1-m/s exhaustive search.

**Supplementary Methods 3.** Water-temperature-based coupling-water sound speed

Water temperature was measured using a UNI-T UT320D thermometer with a K-type thermocouple probe. The meter has a display resolution of 0.1 °C and a manufacturer-specified accuracy of ±(0.5% of the reading + 1 °C) over the relevant temperature range, excluding thermocouple-probe error. The coupling-water sound speed was calculated using the k-Wave waterSoundSpeed implementation of the fifth-order polynomial reported by Marczak (J. Acoust. Soc. Am. 102(5), 2776-2779, 1997; doi:10.1121/1.420332).

c(T) = 2.787860 × 10⁻⁹T⁵ − 1.398845 × 10⁻⁶T⁴ + 3.287156 × 10⁻⁴T³ − 5.779136 × 10⁻²T² + 5.038813T + 1402.385,

where T is the water temperature in °C and c is the sound speed in m/s. The polynomial is valid from 0 to 95 °C. The calculated values were 1492.18 m/s at 23.3 °C for the phantom experiment and 1486.69 m/s at 21.4 °C for the mouse experiment; the manuscript reports these values as 1492 and 1487 m/s, respectively.

**Supplementary Results 3.**

Figure S3 shows the water-temperature-sound-speed relationship over 0-40 °C and enlarges the experimental temperature range. The two measured temperatures lie close on the same smooth curve, and the plotted labels use integer sound speeds consistent with the supported reporting precision.

**Fig. S3.** Coupling-water sound speed calculated from measured temperature. The curve was generated with the k-Wave waterSoundSpeed implementation of the fifth-order Marczak polynomial over 0-40 °C; the polynomial is valid from 0 to 95 °C. Markers denote the measured mouse (21.4 °C; 1487 m/s after rounding) and phantom (23.3 °C; 1492 m/s after rounding) water temperatures. The inset enlarges the experimental range.
